# Extreme point and halving edge search in abstract order types

**DOI:** 10.1016/j.comgeo.2013.05.001

**Published:** 2013-10

**Authors:** Oswin Aichholzer, Tillmann Miltzow, Alexander Pilz

**Affiliations:** a Institute for Software Technology, Graz University of Technology, Austria; b Institute of Computer Science, Freie Universität Berlin, Germany

**Keywords:** Convex hull, Halving line, Abstract order type, CC System, Chirotope

## Abstract

Many properties of finite point sets only depend on the relative position of the points, e.g., on the order type of the set. However, many fundamental algorithms in computational geometry rely on coordinate representations. This includes the straightforward algorithms for finding a halving line for a given planar point set, as well as finding a point on the convex hull, both in linear time. In his monograph *Axioms and Hulls*, Knuth asks whether these problems can be solved in linear time in a more abstract setting, given only the orientation of each point triple, i.e., the setʼs chirotope, as a source of information. We answer this question in the affirmative. More precisely, we can find a halving line through any given point, as well as the vertices of the convex hull edges that are intersected by the supporting line of any two given points of the set in linear time. We first give a proof for sets realizable in the Euclidean plane and then extend the result to non-realizable abstract order types.

## 1 Introduction

In computational geometry, many fundamental properties of finite point sets do not depend on the actual coordinates of each point in real space, but rather on the relative position of the points among each other. In their landmark paper, Goodman and Pollack [1] capture this idea by defining the order type of a point set. In the plane, two point sets have the same *order type* if there is a bijection *π* between the sets s.t. for every triple p,q,r of the first set, the corresponding points π(p),π(q), and π(r) have the same orientation (i.e., are both oriented clockwise or counterclockwise).^3^ This orientation can be tested by the inequality

$$\det\left(\begin{array}{ccc} p_{x} & p_{y} & 1\\ q_{x} & q_{y} & 1\\ r_{x} & r_{y} & 1 \end{array}\right)>0,$$

which indicates whether *r* is to the left of the directed line through *p* and *q*, i.e., whether the triple is oriented counterclockwise. The sign of the determinant therefore gives a predicate ∇(p,q,r) that is true iff the triple is oriented counterclockwise. This mapping of all triples of a set to their orientation is also called the *chirotope* of the set (cf. Remark 1.6 in [2] and [3, p. 95] for details on that term). Many combinatorial properties of a set of points only depend on its order type, like its convex hull, the set of its crossing-free graphs, etc. We implicitly assume throughout this paper that all sets are in general position, i.e., do not contain collinear triples.

In contrast to these properties, there are further, more “metric” properties of a point set that are not determined by the order type. This includes the setʼs Delaunay triangulation; it is straightforward to construct two sets of the same order type whose Delaunay triangulations are different. Nevertheless, the problem can still be considered as being discrete. Guibas and Stolfi [4] separate topological from geometric aspects, using a predicate InCircle(p,q,r,s) that is true iff the triple (p,q,r) is oriented counterclockwise and the point *s* lies inside the circle defined by the first three points. This predicate is equivalent to

$$\det\left(\begin{array}{cccc} p_{x} & p_{y} & p_{x}^{2}+p_{y}^{2} & 1\\ q_{x} & q_{y} & q_{x}^{2}+q_{y}^{2} & 1\\ r_{x} & r_{y} & r_{x}^{2}+r_{y}^{2} & 1\\ s_{x} & s_{y} & s_{x}^{2}+s_{y}^{2} & 1 \end{array}\right)>0.$$

Their Delaunay triangulation algorithm depends almost entirely on this predicate, making it a robust approach, that is intended to be easy to implement and to prove.

Motivated by this approach, Knuth [3] develops axiomatic systems following these two tests. He defines five axioms over a ternary predicate *P* and calls sets of triples obeying them *CC Systems*.

Axiom 1 **cyclic symmetry**

P(p,q,r)⇒P(r,p,q) *.*

Axiom 2 **antisymmetry**

P(p,q,r)⇒¬P(p,r,q) *.*

Axiom 3 **nondegeneracy**

P(p,q,r)∨P(p,r,q) *.*

Axiom 4 **interiority**

P(t,p,q)∧P(t,q,r)∧P(t,r,p)⇒P(p,q,r) *.*

Axiom 5 **transitivity**

P(p,q,r)∧P(p,q,s)∧P(p,q,t)∧P(p,r,s)∧P(p,s,t) ⇒P(p,r,t) *.*

These are fulfilled by all point sets in the Euclidean plane, with P=∇ defined as point triple orientation. For these sets, the CC Systems are equivalent to the point set order types. However, there exist CC Systems that cannot be constructed as point sets in $R^{2}$. These are called *non-realizable* systems, see Section 3.2. CC Systems are equivalent to *abstract order types*, which, for example, can be used in so-called *abstract order type extension* [5]. Every abstract order type can be mapped to an arrangement of pseudo-lines in the projective plane. A stretchable arrangement corresponds to a set of realizable order types [3, pp. 34–35]. Goodman and Pollack showed that all CC Systems of up to 8 elements are realizable as point sets [6]. This fact is useful to show properties of small sets by geometric reasoning.

Theorem 1 **Goodman, Pollack**

*Any arrangement of eight pseudo-lines is stretchable.*

The concept of the convex hull of a point set generalizes to all CC Systems. The axiomatic approach can also be extended to cover Delaunay triangulations. In the axiomatic settings, Knuth provides O(nlogn) time algorithms for both problems, where the time bound for the latter holds in the expected case. He points out that the algorithm of Guibas and Stolfi uses the coordinate representation to find a line that partitions the point set into two equally sized subsets (cf. [4, pp. 110–111]). Open Problem 1 in [3, pp. 97–98] therefore asks for an algorithm to find such a partition of a CC System in linear time. The problem is straightforward when given an extreme point of the set (i.e., an element of the convex hull boundary). Proving the existence of a linear-time algorithm for finding a single extreme point is also explicitly part of Open Problem 1.

In this work, we answer both parts of the open problem in the affirmative. In Section 2, we give a simple O(n) time algorithm that, given a point *c* of a set *S* of size *n*, finds a halving edge through *c*; more specifically, it finds a second point $c^{\prime }\in S$ s.t. not more than $\left\lceil \frac{n-2}{2}\right\rceil$ points are on each side of the supporting line of *c* and $c^{\prime }$. We then describe in Section 3 an algorithm that, given two points *p* and *q*, returns the edge of the convex hull that is crossed by the ray from *p* through *q*. We first show that the algorithm runs in O(n) time for realizable sets. We then show that the time bound is also correct for non-realizable sets, that is, for all CC Systems. Both algorithms use a prune and search approach.

Our main motivation is to show that the asymptotic running time for solving these problems does not depend on the representation by coordinates. While an arbitrary halving edge can easily be found by picking a point with median, say, *x*-coordinate, the problem is more sophisticated when the halving line should pass through a predefined point. E.g., the linear time Ham-Sandwich-Cut algorithm of Lo, Matoušek and Steiger [7] can be adapted to find a halving line through a point. The straightforward way of finding an extreme point of a set given by coordinates is selecting the one with, say, lowest *x*-coordinate. Finding a convex hull edge that is traversed by a given line in linear time is a subroutine of the so-called Ultimate Convex Hull Algorithm of Kirkpatrick and Seidel [8]. There, the median of the slopes of an arbitrary matching of the points is used for the prune and search approach. Recent algorithms that operate only on triple orientations involve computing the convex hull of disks [9]. Also note that there are models that allow for more abstract order types to be realized than by point sets in the Euclidean plane [10].

A preliminary version of this work appeared as [11].

## 2 Computing a halving edge in linear time

In this section we describe an algorithm to compute a halving edge through a given point *c* of a finite point set *S* of size *n* in the plane. While we give the algorithm for realizable order types, note throughout the proof that all assumptions also hold in the abstract setting. The idea is to split the point set evenly by two double wedges centered at *c*. We identify the double wedge that must contain a halving edge, and continue with the points therein.

**Fig. 1:**
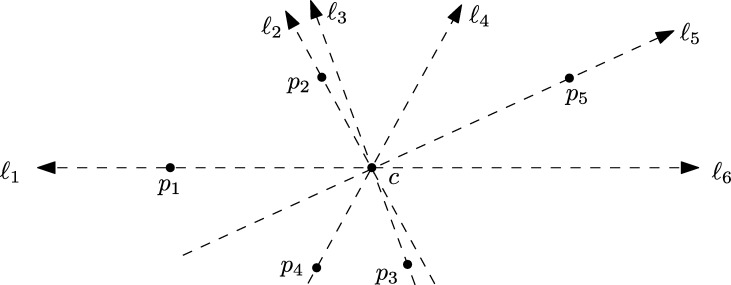
Rotating the line *ℓ* around *c* starting at $p_{1}$ induces an order on the set.

We start by picking a point $p_{1}\in S\setminus \{c\}$ arbitrarily. Imagine a directed line *ℓ* rotated by 180° clockwise around *c*, starting at $p_{1}$. Let $p_{2},\ldots ,p_{n-1}$ be the remaining points in the order *ℓ* passes through them, either with its front or its tail. We denote this order by ≺, which is actually the order described in [3, p. 16]. By $\ell_{1},\ldots ,\ell_{n}$ we denote the “snapshots” of *ℓ* at the points $p_{1},\ldots ,p_{n-1},p_{1}$ respectively. Thus, $\ell_{1}$ and $\ell_{n}$ have opposite directions. See Fig. 1 for an illustration.

**Fig. 2:**
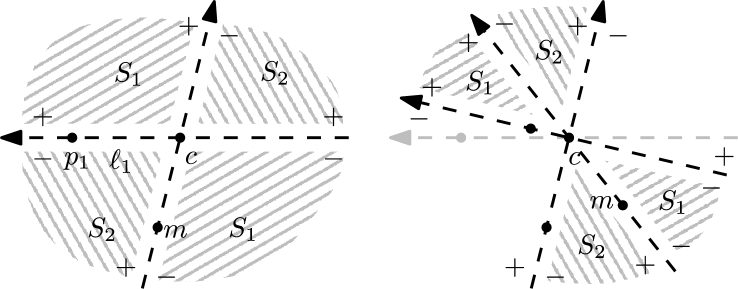
The sign + marks the half plane which contains more than $\frac{n-2}{2}$ points. $S_{1}$ and $S_{2}$ are indicated by the gray tiling patterns. The figure shows the first (left) and a later iteration (right).

For each pair of S∖{c}, we can compute the relative order in which *ℓ* passes through them in constant time, using the predicate ∇. Thus the median $m=p_{\left\lfloor \frac{n}{2}\right\rfloor }$ with respect to ≺ can be computed in O(n) time [12]. (Note that the number of points to the right of *ℓ* may *not* change monotonically while rotating.) The supporting lines of $cp_{1}$ and *cm* define two double wedges, containing $S_{1}=\{p\in S\setminus \{c\}:p≺m\}$ and $S_{2}=\{p\in S\setminus \{c\}:m≺p\}$, respectively, each having not more than $\left\lceil \frac{n-2}{2}\right\rceil$ points. See Fig. 2. We use the following standard argument. If $\ell_{1}$ is not a halving line, we have w.l.o.g. more than $\left\lceil \frac{n-2}{2}\right\rceil$ points to the right of $\ell_{1}$ and thus less than $\left\lfloor \frac{n-2}{2}\right\rfloor$ points to the right of $\ell_{n}$. Due to our general position assumption, while rotating *ℓ* only one point changes sides at the same time. Thus, *ℓ* must be a halving line at some point during the rotation. Assume that $\ell_{\left\lfloor \frac{n}{2}\right\rfloor }$ is not a halving line. If less than $\left\lfloor \frac{n-2}{2}\right\rfloor$ points are right of $\ell_{\left\lfloor \frac{n}{2}\right\rfloor }$, then one of the points in $S_{1}$ forms a halving edge with *c* (recall that there are w.l.o.g. more points right of $\ell_{1}$). Otherwise one of the points in $S_{2}$ does.

This way we can decide which subset contains a halving edge, exclude the other subset and iterate. Observe that all the points we exclude belong to two (open) wedges centered at *c*, one entirely to the left and one entirely to the right of the non-removed lines of $\ell_{2},\ldots ,\ell_{n-1}$. The closure of each wedge contains a ray of $\ell_{1}$. We store how many points each of these wedges contained. Now, if we want to compute the number of points to the right of some line $\ell_{i}$ in subsequent iterations, then we only need to consider the remaining points and add the number of points belonging to the excluded wedge to the right of $\ell_{i}$. The algorithm has linear running time, as we exclude approximately half of the points in each linear-time iteration.

Theorem 2 *Given a point set*$S\subset R^{2}$*of size n in general position and a point*c∈S*, a halving edge of S incident to c can be found in linear time using only the predicate* ∇*.*

Now we argue that the above approach does not require that *S* is realizable, that is, it works for any CC System (using the predicate *P*). The order ≺ around *c* we use is also defined for CC Systems by signing the points in a way that the positive points are to the right of $\ell_{1}$ and the negative ones are to the left of $\ell_{1}$, where each negative sign will change the orientation of a triple containing *c* [3, p. 16] (observe that all our orientation tests include *c*). This allows us to give a purely combinatorial formulation of our algorithm: Let *s* be a variable initialized to 0 and let $r_{i}$ be the number of negative points preceding point $p_{i}$ plus the number of positive points succeeding $p_{i}$ with respect to ≺ in the current iteration (this corresponds to the points to the right of $\ell_{i}$). We may assume that initially we have $r_{1}>\frac{n-2}{2}>r_{n}$. If the sum of $r_{m}$ and *s* is less than $\left\lfloor \frac{n-2}{2}\right\rfloor$, we remove the points that succeed *m*, and add the number of removed positive points to *s*. Otherwise, we remove the points that precede *m* and add the number of removed negative points to *s*. Note that for every point $p_{i}$, the value of $r_{i}+s$ is an invariant over all iterations. Eventually, we end up with an element $p_{j}$ such that $r_{j}+s=\lfloor \frac{n-2}{2}\rfloor$.

In the combinatorial formulation of the algorithm, the crucial properties of the sequence *r* we use is that subsequent elements change by one and that the required value is between $r_{1}$ and $r_{n}$. Therefore, the algorithm is also adaptable to more sophisticated problems than finding a halving edge. One example is the search for a *balanced line*, that is, a line that separates a set consisting of *n* white points and *n* black points in a way that the difference between the black and white points on each side of the line is 0 (additional care has to be taken when defining the sequence *r* since a balanced line does in general not pass directly through a given point *c*, i.e., there is no explicit representation of the line as a supporting line of two points). An application of this can be found in [13]. There, it is shown that certain crossing-free matchings on a point set are not unique by constructing a balanced line crossing a segment of a given matching.

For realizable sets, the algorithm can directly be extended to higher dimensions. Order types in $R^{d}$ are defined analogously as in the plane, see [1]. The orientation of a (d+1)-simplex can be computed by the corresponding determinant. Let $\left(p_{1},\ldots ,p_{d+1}\right)$ be an ordered tuple of points in $R^{d}$. For the oriented hyperplane *H* through the points $\left(p_{1},\ldots ,p_{d}\right)$, $\nabla (p_{1},\ldots ,p_{d+1})$ indicates on which side of *H* the point $p_{d+1}$ lies.

Corollary 3 *Given a point set*$S\subset R^{d}$*in general position, d constant, and distinct points*$c_{1},\ldots ,c_{d-1}\in S$*, one can find a halving hyperplane through*$c_{1},\ldots ,c_{d-1}$*in linear time using only* ∇*.*

## 3 Computing a convex hull edge in linear time

In this section we show how to compute a convex hull edge of a set in linear time, only using the orientation of triples. We first present the algorithm for point sets and then extend it to general CC Systems.

### 3.1 Realizable point sets

**Fig. 3:**
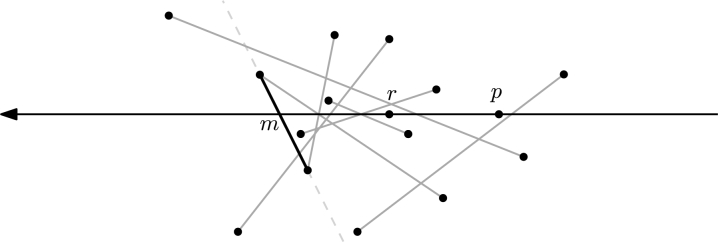
An arbitrary matching of edges that partition *S*, and *m*, the result of BasicMin.

As a first step we describe an algorithm called BasicMin, which plays a crucial role as a subroutine. Let *S* be a point set in the plane and suppose we are given two points p,r∈S. We assume that *pr* is a halving edge of *S* and that n=|S| is even. W.l.o.g. let *r* be the coordinate origin and let *p* be on the positive part of the *x*-axis. Let *M* be an arbitrary perfect matching between the points above and below the *x*-axis, i.e., for any edge s=ab∈M we have ∇(p,r,a)≠∇(p,r,b). See Fig. 3 for an illustration.

Let ⋉ be the binary operator that accepts two edges $s,s^{\prime }\in M$ as input and returns the edge on the convex hull boundary of $s\cup s^{\prime }$ that crosses the *x*-axis at the smallest *x*-coordinate, i.e., the pair of endpoints whose upward-directed supporting line has all other points of *s* and $s^{\prime }$ to the right. The relevant property of the operator is that the crossing of $⋉(s,s^{\prime })$ with the *x*-axis is not to the right of the crossings of *s* and $s^{\prime }$ with the *x*-axis.

BasicMin takes a point set *S* and two points *p* and *r* as input, partitions *S* arbitrarily into the matching $M=\{s_{1},\ldots ,s_{\left(\frac{n-2}{2}\right)}\}$, and computes a special edge $m=m_{\left(\frac{n-2}{2}\right)}$ iteratively via

$$m_{1}=s_{1},$$

$$m_{\left(i+1\right)}=⋉(m_{i},s_{\left(i+1\right)}).$$

Obviously, the running time of BasicMin is linear in *n*. Note that *m* does not need to be on the convex hull of the whole set. Also, *m* may depend on the (undefined) order in which the elements of *M* are processed by BasicMin. However, *m* has the following useful property.

Lemma 4 *Let ℓ be a line directed upwards, crossing the x-axis left of the crossing of m with the x-axis. Then at least* $\frac{n-2}{2}$ *points of S are to the right of ℓ.*

Proof Note that the crossing of *ℓ* with the *x*-axis is further to the left than any other such crossing of *M*. Assume, for the purpose of contradiction, that there is an edge in *M* for which both points lie to the left of *ℓ*. Then also the crossing with the *x*-axis is to the left of the crossing of *m*, a contradiction. Hence, for each of the $\frac{n-2}{2}$ edges in *M* at least one endpoint lies to the right of *ℓ*.  □

Note that, even though the argumentation of Lemma 4 involves relative positions of crossings, the constant-time operation ⋉ can be expressed by using only ∇. Now we are ready to give the main algorithm.

Theorem 5 *Given two points*p,q*of a point set*$S\subset R^{2}$*of size n in general position, one can find the edge e of the setʼs convex hull that passes through the ray pq in*O(n)*time using the predicate* ∇*.*

Proof W.l.o.g. let *pq* be horizontal with *q* left of *p*. Note first that the case where *q* is a vertex of the convex hull can be identified in linear time. We therefore concentrate on the setting where one endpoint of *e* is below *pq* and the other one is above. Let *U* be the set strictly above *pq*, whereas *L* is the set strictly below *pq*. W.l.o.g. let $|U|⩾\frac{n-2}{2}$.

**Fig. 4:**
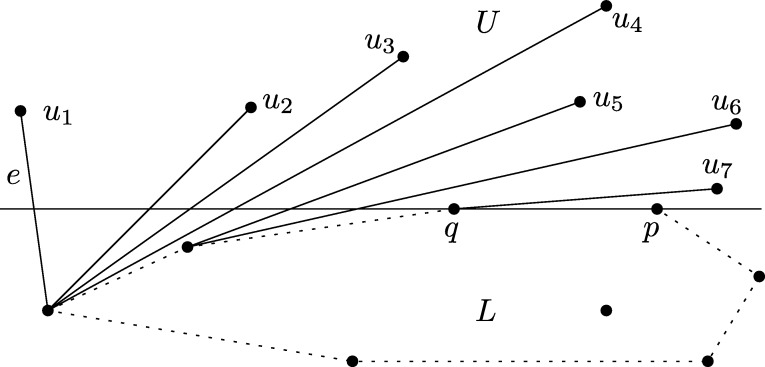
The order on *U* defined by removing vertices of the intersected convex hull edge.

Consider the endpoint $u_{1}\in U$ of *e*. If we remove $u_{1}$, the ray *pq* intersects the boundary of the (new) convex hull at a new edge $e^{\prime }$ with an endpoint $u_{2}\in U$. Note that the other endpoint of $e^{\prime }$ might now be *q*. In any case, iteratively removing points from *U* of the intersected convex hull edge induces an order on *U* (see Fig. 4). Note that this corresponds to the order in which the points of *U* are traversed by a tangent *t* of CH(L∪{p,q}) that is rotated clockwise around that hull, starting at *e* and ending at *pq*. The main observation is that in the search for $u_{1}$, given a point $u_{i}$ and the tangent *t* passing through $u_{i}$, we can discard all points of *U* to the right of $u_{i}$ with respect to the point *l* where *t* touches CH(L∪{p,q}), since none of these points can be $u_{1}$. The support l∈L∪{q} of *t* can be found in linear time, since the radial order of L∪{p,q} around any $u_{i}$ is linear.

Note that these observations already imply the following randomized approach. Select any element $u_{i}$ of *U* at random. The other support *l* of the tangent (recall that this might as well be *q*) can be found in linear time. We discard the points of *U* right of $lu_{i}$ and iterate. However, consecutive “bad” choices of $u_{i}$ result in overall quadratic worst-case behavior. We therefore have to make a “good” choice of $u_{i}$ in order to discard a linear number of points per iteration.

**Fig. 5:**
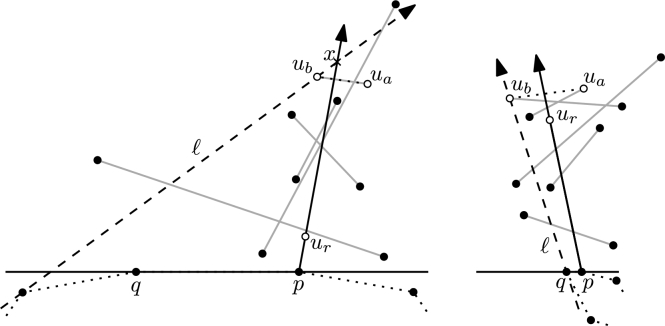
The edge $u_{a}u_{b}$ allows to prune half of the upper points.

The points of *U* are ordered linearly around *p*. Let $u_{r}$ be the median of this order, which we select in linear time. Let *M* be an arbitrary perfect matching between the points of *U* to the left and to the right of $pu_{r}$ (maybe omitting one point), see Fig. 5. Now we apply BasicMin on *M* with $r=u_{r}$, which results in an edge $m=u_{a}u_{b}$. By construction, all edges of *M* as well as $u_{a}u_{b}$ cross the ray $pu_{r}$. Now, find the tangents $t_{a}$ and $t_{b}$ of CH(L∪{p,q}) through $u_{a}$ and $u_{b}$, respectively (we consider them being directed upwards). Let $\ell =t_{a}$ if $u_{b}$ is to the right of $t_{a}$, otherwise let $\ell =t_{b}$. Note that *p* is right of *ℓ* since *q* is included in the set that we place the tangent on. There are two cases to consider. If *ℓ* crosses the ray $pu_{r}$ at a point *x*, then the crossing of $u_{a}u_{b}$ is on the ray between *p* and *x* (by definition of *ℓ*). Due to Lemma 4, at least half of the points of *M* are to the right of *ℓ*. Otherwise, if *ℓ* does not cross the ray $pu_{r}$, then all points of *U* to the right of the ray are also to the right of *ℓ*. In both cases, we can discard at least half of the points, which is at least a quarter of the overall set *S* (recall that *U* was w.l.o.g. larger than *L*; in each iteration, the process is applied to the larger of the two sets). We can therefore in linear time reduce this problem to constant size such that it then can be solved by a brute-force approach.  □

Note that the transitivity of the order on *U* directly follows from the definition of the convex hull and carries over to general CC Systems. Also, the transitivity of L∪{p,q} around any point of *U* holds for CC Systems due to Axiom 5. This already implies that the algorithm is correct for any CC System. However, the linear time bound depends on the number of points that are to the right of *ℓ*. In order to show that the bound also holds for abstract order types, we need to prove that BasicMin also works as expected on non-realizable sets, and that then also *ℓ* has at least half of the points of *U* to the right.

### 3.2 Non-realizable sets

**Fig. 6:**
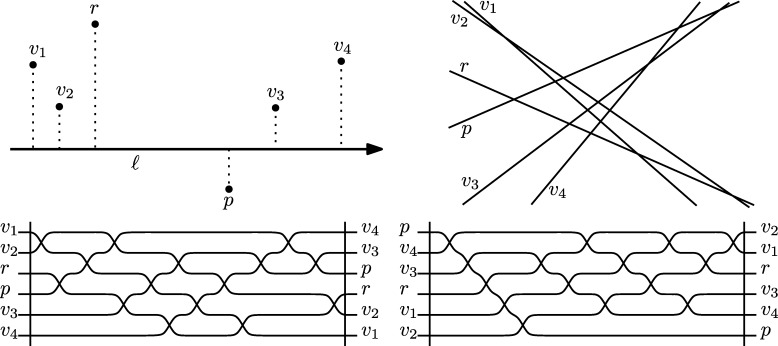
From a point set and its circular sequence (top left) to its dual line arrangement (top right) and a corresponding pseudo-line arrangement (bottom left). The transformed arrangement to the bottom right represents the same order type.

There exist several equivalent definitions for the convex hull of a finite point set *S* in the plane. For example, the convex hull can be defined as the convex polygon of smallest area that contains all points of the set. Equivalently, the boundary of the convex hull of *S* is the sequence of pairs *uv* with {u,v}⊂S such that all points S∖{u,v} are to the left of *uv*. This latter definition is more combinatorial and carries over to the abstract setting, where we are no longer concerned with “geometric artefacts” like area or perimeter.^4^ In this subsection, we will give a brief summary of the relevant properties of abstract order types, following Goodman and Pollack [2], which we suggest to the reader for further information on that topic, in addition to [3]. Throughout this section, see Fig. 6 for illustrations.

Let us again start in the Euclidean plane by considering a point set *S*, which we still require, for ease of presentation, to be in general position (see [2] on how to handle other cases). Let *ℓ* be a directed line not orthogonal to any supporting line of *S*. Project the points orthogonally on *ℓ*, which gives us a sequence of the points along *ℓ*. If we rotate *ℓ* in counterclockwise direction, then eventually two points, let them be *u* and *v*, will happen to be projected to the same point on *ℓ*. When continuing the rotation process, *u* and *v* will have changed their position in the sequence along *ℓ*. After a rotation of 180°, the initial sequence of the projected points on *ℓ* will be reversed. Continuing the rotation gives an infinite periodic sequence of transpositions like the one of *u* and *v*. It is called the *circular sequence of permutations associated to S*, or the *circular sequence* of *S*, for short. One can observe that after a reversal of *u* and *v* all other pairs are reversed until *u* and *v* are reversed again.

The circular sequence allows us to observe several geometric properties of *S*. A point that precedes all others in some permutation (on *ℓ*) in the circular sequence is an extreme point. Note that all the information we can get from the circular sequence is encoded in one half-period. We therefore can identify the vertices of the convex hull as those points that are topmost or bottommost on *ℓ* at some point, when we look at *ℓ* such that it is directed downwards. For every subset of *S*, the circular sequence therefore gives the point on its convex hull. Consider again the moment when *u* and *v* change their position in the permutation, say from *uv* to *vu*. Then the triple *uvw* is oriented counterclockwise if and only if *w* is projected below (i.e., after) both *u* and *v* when they change their position (assuming counterclockwise rotation of *ℓ*). Note that this requires that at the beginning of the half-period *u* is above (before) *v*. It is common and convenient to identify the points of *S* with their order at the initial position of *ℓ*. For $S=\{p_{1},\ldots ,p_{n}\}$, we therefore start with $p_{i}$ above $p_{j}$ on *ℓ* iff i<j. While the starting position of *ℓ* in the half-period is arbitrary, this labeling is useful when comparing point triples. After a half-period, the points are given in the sequence $\langle p_{n},\ldots ,p_{1}\rangle$.

The circular sequence contains strictly more information than the order type of a set. While the order type is implied by the circular sequence, it is rather straightforward to construct point sets with different circular sequences that have the same order type.

Consider an arrangement of pseudo-lines in the projective plane $P^{2}$ (a set of closed curves not separating $P^{2}$ that pairwise intersect in exactly one point). An arrangement is *simple* if no three pseudo-lines intersect in one point. One important property of pseudo-line arrangements, shown by Levi [14], is that they can be extended by additional pseudo-lines.

Lemma 6 **Levi Enlargement Lemma**

*Given a pseudo-line arrangement*A*in*$P^{2}$*and two points that do not both lie on the same element of*A*, there exists a pseudo-line arrangement*A∪{χ}*such that the pseudo-line χ passes through these two points.*

Using this lemma, choose a pseudo-line $\ell_{\infty }$ not part of the arrangement as the line at infinity. Place a point *ψ* on $\ell_{\infty }$. If we choose *ψ* as the point at vertical infinity, we can identify the arrangement of pseudo-lines with an arrangement of *x*-monotone pseudo-lines in the Euclidean plane (where the curves are no longer closed). This requires the application of the Levi Enlargement Lemma to get *x*-monotonicity, details are given in [2]. If we sweep this arrangement from left to right, we get again a half-period of a circular sequence, where a change of a pair happens as we sweep across the intersection of two pseudo-lines. Goodman and Pollack [2] show that any circular sequence of a point set describes the topology of an arrangement of pseudo-lines in $P^{2}$. Actually, for every point set in the Euclidean plane, the standard duality transform for point sets gives an arrangement of straight lines whose crossings, when sweeping from left to right, give a half-period of the circular sequence of the set [15]. There are, however, arrangements of pseudo-lines in the projective plane that are not stretchable. For them, no point set in the Euclidean plane exists that implies any sequence derived from such an arrangements by any choice of *ψ*.

Recall that we were able to derive the orientation of a point triple *uvw* by the position of the points in a half-period of the circular sequence. Given a direction around a point at infinity allows us to give a triple of pseudo-lines an orientation, even if there does not exist a corresponding point set order type in the Euclidean plane (i.e., the sequence is non-realizable); we still get a predicate $\nabla_{a}$ that gives each triple an orientation, that is, an *abstract order type*. Knuth [3] shows that, for any simple arrangement, $\nabla_{a}$ fulfills the five axioms defining CC Systems, and from any CC System a corresponding arrangement can be derived; hence, the terms “CC System” and “abstract order type” are merely synonyms. We can use either the axioms or a pseudo-line arrangement (together with a direction around a point at infinity) for arguing about abstract order types. In the next section, we will use the model of *x*-monotone pseudo-lines in the Euclidean plane.

At first sight, the identification of abstract order types with pseudo-line arrangements in the projective plane might feel like a detour to *x*-monotone arrangements in the Euclidean plane as representation of circular sequences. This might be the case for the application of the concept in this paper, however, it is profound in the whole theory about abstract order types. Recall that different circular sequences might correspond to two point sets having the same order type. This is also the case for non-stretchable arrangements. (On the other hand, for arrangements of pseudo-lines in the projective plane, there are transformations that are order-type-preserving and some that are not.) If two pairs *uv* and *st* of four different points change their position in the sequence and *uv* happens right before *st* in the half-period, then changing *st* right before *uv* results in the same order type. (To reduce the cases to consider, we will nevertheless make use of that relative position of pseudo-line crossings in the next section.) There is another aspect that is not directly captured by *x*-monotone arrangements in the Euclidean plane. When mapping the arrangement from the projective to the Euclidean plane, we chose the line at infinity through *ψ* arbitrarily; however, the topology of the arrangement in the projective plane does not depend on the choice of this line. Analogously, the circular sequence of a point set does not depend on the actual starting position of *ℓ*. In terms of pseudo-lines in the Euclidean plane, the following can be shown (more details are given in [2], [3]). Consider a crossing of two pseudo-lines that has no further crossings to the left from its initial position. Then we can move this crossing to the right end of the arrangement, such that the resulting arrangement still represents the same abstract order type. In other words, we “untangle” the crossing at the left end and introduce a new one to the right (note that this corresponds to removing the position change *uv* at the beginning by a change *vu* at the end). For every abstract order type and any crossing therein, we can therefore choose a *x*-monotone representation where that crossing is leftmost.

### 3.3 A general proof of the time bound

To see why care has to be taken when we deal with non-realizable order types, note that in general the order type does not capture the relative position of the supporting lines of point pairs and the crossings of these lines. However, abstract order types capture some of the information that is related to crossings of supporting lines, which allows us to show that the properties needed for BasicMin are also present in the abstract setting.^5^ We use the dual representation of abstract order types in the Euclidean plane by *x*-monotone pseudo-lines; this allows us to use the obvious meaning of terms like “above” and “below” when describing an arrangement A (e.g., a pseudo-line *a* is above a point *b* when the point on *a* that has the same *x*-coordinate as *b* has a larger *y*-coordinate than *b*).

Let us recall the problem setting. We are given a set *S* of *n* elements (which we call points, even though *S* might not be a realizable point set), containing two special points *p* and *q*. The set *S* is separated by *pq* into a set *U* to the right of *pq*, and a set *L* to the left of it (where “left” and “right” are indicated by a predicate $\nabla_{a}$). We want to obtain the pair $l_{1}u_{1},l_{1}\in L,u_{1}\in U$ that is consecutive on the convex hull of *S*, where *p* is to the right of $l_{1}u_{1}$. This is done by obtaining a pair $\ell =l_{j}u_{i}$ such that at least half of the points of *U* (minus a constant) are to the right of *ℓ* and no point of *L* is to the left of *ℓ*.

Consider the dual *x*-monotone pseudo-line arrangement A representing the abstract order type of *S*. Keep in mind that *p* is an extreme point of the set U∪{p}, and that $r=u_{r}$ is the median of the points in *U* ordered radially around *p*. We can represent the arrangement such that the crossing of the pseudo-lines *p* and *r* (which corresponds to the supporting line of *pr* in the primal^6^) is the leftmost crossing in the *x*-monotone representation of A. The linear order of the points around *p* in the primal splits the set $U\setminus \{u_{r}\}$ into *left* and *right* points, separated by *pr*. In the dual, the right pseudo-lines pass above the crossing *pr*, and the left pseudo-lines pass below. Recall the description of BasicMin. *M* is an arbitrary perfect matching between the left and the right points. The operator ⋉ accepts two point pairs, each pair consisting of a left and a right point. The output of the operator is a pair *z* consisting of a left and a right point such that all other points are to the right of the oriented line through these points. This pair *z* is well-defined in the abstract setting as well (and there is always a geometric representation due to Theorem 1). Recall that we compute a special pair $m=m_{\left(\frac{n-2}{2}\right)}$ iteratively via

$$m_{1}=s_{1},$$

$$m_{\left(i+1\right)}=⋉(m_{i},s_{\left(i+1\right)}).$$

**Fig. 7:**
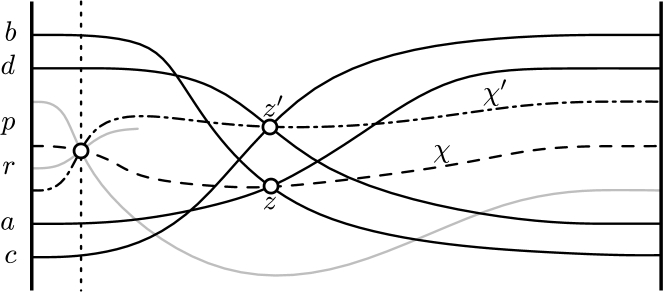
Observation 7: A pseudo-line $\chi^{\prime }$ (not part of the arrangement) witnesses that no element is left of $z^{\prime }$ in the primal.

The crucial property of the pair $m=u_{a}u_{b}$ (and later the line *ℓ* tangent to CH(L∪{p,q}) through $u_{a}$ or $u_{b}$) is that at least one endpoint of each pair $s_{i}\in M$ lies on the same side of *m* as the pivot *p*. (We assume that *m* and the elements of *M* are directed from the left point to the right point and hence *p* is to the right of *m*.) In the dual, each element of *M* is represented as the crossing of a right and a left pseudo-line in A (this corresponds to the supporting line of the matched pair of points in the primal). The crucial property for *m* in the dual therefore is to have at least one pseudo-line of each pair $s_{i}\in M$ passing below its crossing in A. For realizable point sets, we were able to argue for the correctness of BasicMin using the intersection *χ* of a matched pair *s* with the supporting line of *p* and *r*. In the dual, this intersection *χ* corresponds again to a pseudo-line that can be added to A; this pseudo-line passes through the crossing *pr* and *s*. Also for non-realizable sets, such a pseudo-line *χ* exists due to the Levi Enlargement Lemma. In fact, in non-realizable settings the intersections behave in the same transitive manner as in the realizable setting; see Fig. 7 for an illustration of the following statement.

Observation 7 *Let*z=(a,b)*and*$z^{\prime }=(c,d)$*be two pairs such that the point p is to the right of both pairs* (*i.e., in the dual the pseudo-line p is below the crossings z and* $z^{\prime }$)*. Let χ* ($\chi^{\prime }$) *be a dual pseudo-line through the crossings pr and z* ($z^{\prime }$)*. If none of a and b is to the left of the primal line* $z^{\prime }$*, then the dual pseudo-line* $\chi^{\prime }$ *is above χ in the part of the dual arrangement that is to the right of the dual crossing pr. This also holds if* a=c *or* b=d*.*

Hence, after applying BasicMin to the matching *M*, we obtain in the dual a pseudo-line crossing *m* on a pseudo-line $\chi_{m}$ that passes through the crossing *pr*, such that no crossing of *M* is above $\chi_{m}$. Suppose, for the sake of contradiction, that there is a pair (a,b) such that both dual pseudo-lines *a* and *b* pass above the crossing *m*, but the crossing *ab* is below $\chi_{m}$. If the crossing *ab* is to the left of *m* in the *x*-monotone arrangement, then, when traversing *a* from left to right, one would have to pass below $\chi_{m}$ and then go above it again before *m*. Otherwise, if the crossing *ab* is to the right of *m*, then *b* would have to intersect $\chi_{m}$ to be above it at *m* and then has to be below $\chi_{m}$ again to reach the crossing *ab*. Both cases contradict the fact that a pair of pseudo-lines intersects exactly once in the *x*-monotone arrangement. Hence, half of the pseudo-lines are below the pseudo-line crossing *m* in A. This corresponds to at least half of the points being on the same side of *m* as *p*.

Recall that in the proof of Theorem 5, we chose a directed line *ℓ* supported by a point l∈L∪{q} and either $u_{a}$ or $u_{b}$ such that no elements of $L\cup \{p,q,u_{a},u_{b}\}$ is to the left of it. We now proceed to show that at least one point of each pair (v,w) in *M* is to the right of the directed line *ℓ*, as demanded in the proof of Theorem 5. The points involved are $\left\{p,q,r,l,u_{a},u_{b},v,w\right\}$, where *l* and *q* might be the same point. Since these are at most eight points, we are allowed to use geometric arguments due to Theorem 1. However, we must not rely on the positions of the crossing points on the ray *pr*. Suppose, for the sake of contradiction, that neither of *v* and *w* is right of *ℓ*. At least one of *v* or *w* has to be to the right of $u_{a}u_{b}$. The line *ℓ* separates *v* and *w* from the remaining subset. Further, *v* and *w* are separated by *pr*. These observations imply that *vw* is an edge of the convex hull of $\left\{p,u_{a},u_{b},v,w\right\}$. However, this means that the crossing of the pseudo-lines *v* and *w* is above $u_{a}$ and $u_{b}$ in A, which contradicts the fact that $u_{a}u_{b}$ is *m*. Thus, we conclude

Theorem 8 Theorem 5*also holds for non-realizable CC Systems, i.e., abstract order types.*

## 4 Conclusion

We presented two algorithms that only use the information whether a point triple is oriented clockwise or counterclockwise. Both, a halving edge through a given point and a convex hull edge crossing a specified ray, can be found in linear time, without being given the coordinate representation. We showed that the algorithms also work for general CC Systems (i.e., abstract order types), and thus answer a long-standing open problem of Knuth [3] in the affirmative.

Note that the parts of the so-called Ultimate Convex Hull Algorithm by Kirkpatrick and Seidel [8] that depend on coordinates are essentially the one that find the convex hull edge on the ray that separates a subproblem into two parts. Also, Chanʼs output-sensitive algorithm [16] can be implemented in our setting using Theorem 5. Both allow to improve the time bound given in [3] for realizable point sets regarding output-sensitivity to O(nlogh) for *h* extreme points.

## Footnotes

3 It is common to also consider sets to be of the same order type if the orientation of all triples is inverted in the second set, i.e., the second set can be seen as a mirrored copy of the first set.

4 While the convex hull actually is a convex set, it is not uncommon to refer to its boundary as the “convex hull” (especially when only concerned with finite point sets, as in [3]).

5 This observation is due to an anonymous referee, a previous version proved these properties using a lengthy case distinction.

6 For simplicity, we use the same name in the primal and the dual.
